# Clinical value of multimodal ultrasound in evaluating intestinal stiffness and fibrosis in active Crohn’s disease

**DOI:** 10.1186/s13244-025-02124-0

**Published:** 2025-11-01

**Authors:** Xielu Sun, Chengfang Wang, Dingyuan Hu, Guolong Ma, Zhihua Xu

**Affiliations:** 1 Department of Ultrasonic Diagnosis, The Second Affiliated Hospital and Yuying Children’s Hospital, Wenzhou Medical University, Wenzhou, China; 2 Wenzhou Key Laboratory of Structural and Functional Imaging, Wenzhou, China; 3 Department of Gastroenterology, The Second Affiliated Hospital and Yuying Children’s Hospital, Wenzhou Medical University, Wenzhou, China

**Keywords:** Crohn’s disease, Multimodal ultrasound, Ultrasonic elastography

## Abstract

**Objective:**

It was hypothesized that virtual touch tissue imaging and quantification (VTIQ) is more accurate in quantifying intestinal stiffness compared to conventional B-mode ultrasound for detecting Crohn’s disease (CD) stenosis. We aimed to explore the diagnostic value of multimodal ultrasound in intestinal stenosis of CD.

**Materials and methods:**

A retrospective analysis of CD patients (May 2020 to October 2024) was conducted. Risk factors associated with intestinal stenosis in CD were identified using univariate and multivariate logistic regression analysis. The area under the curve (AUC) of the receiver operating characteristic (ROC) of combined indices was compared with individual indices to assess their predictive ability for intestinal stenosis in CD.

**Results:**

Sixty-three patients were included. Univariate and multivariate logistic regression analysis identified shear wave velocity (OR = 3.943, *p* = 0.008), Young’s modulus value (OR = 1.079, *p* = 0.046), and intestinal bowel ultrasound stenosis assessment score (IBUS-SAS; OR = 1.033, *p* = 0.015) as significant risk factors. The AUC for IBUS-SAS was 0.671, for shear wave velocity was 0.838, and for Young’s modulus value was 0.788. The combined model yielded an AUC of 0.878. Compared to the gold standard (Simplified Endoscopy for Crohn’s Disease, SES-CD), the ultrasound-based approach showed 100% specificity and 71% sensitivity for stenosis detection.

**Conclusion:**

IBUS-SAS, shear wave velocity, and Young’s modulus were independent risk factors for CD intestinal stenosis, with shear wave velocity being the most accurate (AUC = 0.838), supporting our hypothesis. These findings warrant validation in large, high-quality studies.

**Critical relevance statement:**

This study examines the potential of VTIQ ultrasound to assess intestinal stiffness in CD, offering a non-invasive, radiation-free approach that may enhance diagnostic capabilities and contribute to clinical radiology practice.

**Key Points:**

- VTIQ non-invasively assesses intestinal stiffness in CD.
- Shear wave velocity, Young’s modulus, and IBUS-SAS predict stenosis.
- Integrated indices improve diagnostic accuracy.
- VTIQ shows promise for safe, non-invasive diagnosis.
- Requires large-scale, multicenter studies for confirmation.

**Graphical Abstract:**

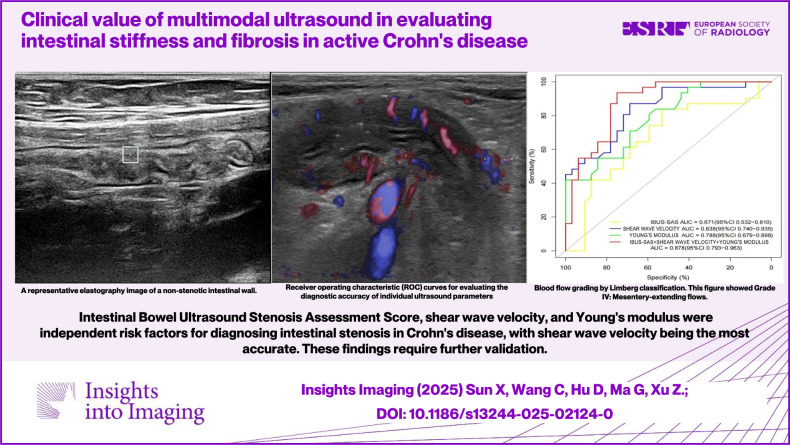

## Introduction

Crohn’s disease (CD) is a chronic, non-specific inflammatory condition primarily characterized by intestinal immune dysfunction. It is marked by a prolonged course, frequent relapses, and poor prognosis [1, 2]. The disease often leads to complications such as fibrosis, strictures, and fistulas, which significantly impair patients’ quality of life [3].

CT and MRI can detect lesions in affected intestinal segments and the intestinal wall; however, CT involves radiation exposure, and MRI is costly, time-consuming, and has numerous contraindications [4, 5]. Gastrointestinal endoscopy provides clear visualization of mucosal changes but cannot comprehensively evaluate the overall structural changes of the intestinal wall and extraintestinal conditions [6, 7]. These limitations necessitate the development of more effective diagnostic methods.

Transabdominal intestinal ultrasound is an emerging, real-time, non-invasive, and reproducible diagnostic tool. Ultrasound elastography can qualitatively or quantitatively assess tissue hardness, offering reliable detection of intestinal fibrosis. However, there is no standardized method for ultrasound elastography in intestinal lesions.

The VTIQ (virtual touch tissue imaging and quantification) technique, a third-generation shear wave imaging technology, has been reported by multiple research groups over the past decade and has been validated in various clinical settings [8, 9]. This technology allows for quantitative assessment of tissue stiffness and includes a quality control (QC) mode to enhance image repeatability and operational independence.

Therefore, this study aims to evaluate the clinical value of VTIQ in quantifying intestinal wall stiffness and to explore the diagnostic potential of multimodal ultrasound in patients with active CD. We hypothesize that VTIQ-derived shear wave velocity and Young’s modulus are superior to conventional ultrasound indices in detecting intestinal stenosis.

## Materials and methods

### Patient source

This study was conducted on 63 patients diagnosed with CD at The Second Affiliated Hospital and Yuying Children’s Hospital of Wenzhou Medical University from May 2020 to October 2024.

### Inclusion and exclusion criteria

#### Inclusion criteria

Primary diagnosis of CD; age > 18 years; complete clinical data, including ultrasonography and enteroscopy; high patient compliance.

#### Exclusion criteria

Severe hepatic or renal insufficiency; cardiovascular disease; other digestive system diseases; cognitive impairment; contraindications to endoscopy or ultrasonography.

### Data collection

Clinical data, including medical history, physical examination, and imaging results, were collected by specialized personnel. All VTIQ examinations were performed on a Mindray Resona R9T system (9L-4 linear transducer, 4–9 MHz); all scans were performed by a single radiologist specialized in intestinal ultrasound, with 7 years of experience and over 80 CD cases evaluated annually. This radiologist had completed a standardized 3-month VTIQ training program.

### Standardized protocols

Preset: Abdominal Bowel Exam Mode (depth 3–8 cm, mechanical index (MI) < 0.7).

Region of interest (ROI): 3 × 3 mm box placed at the thickest bowel wall (> 3 mm).

QC: Daily phantom calibration (Mindray E-1, shear wave elastography (SWE) error < 0.3 m/s).

**Fig. 1:**
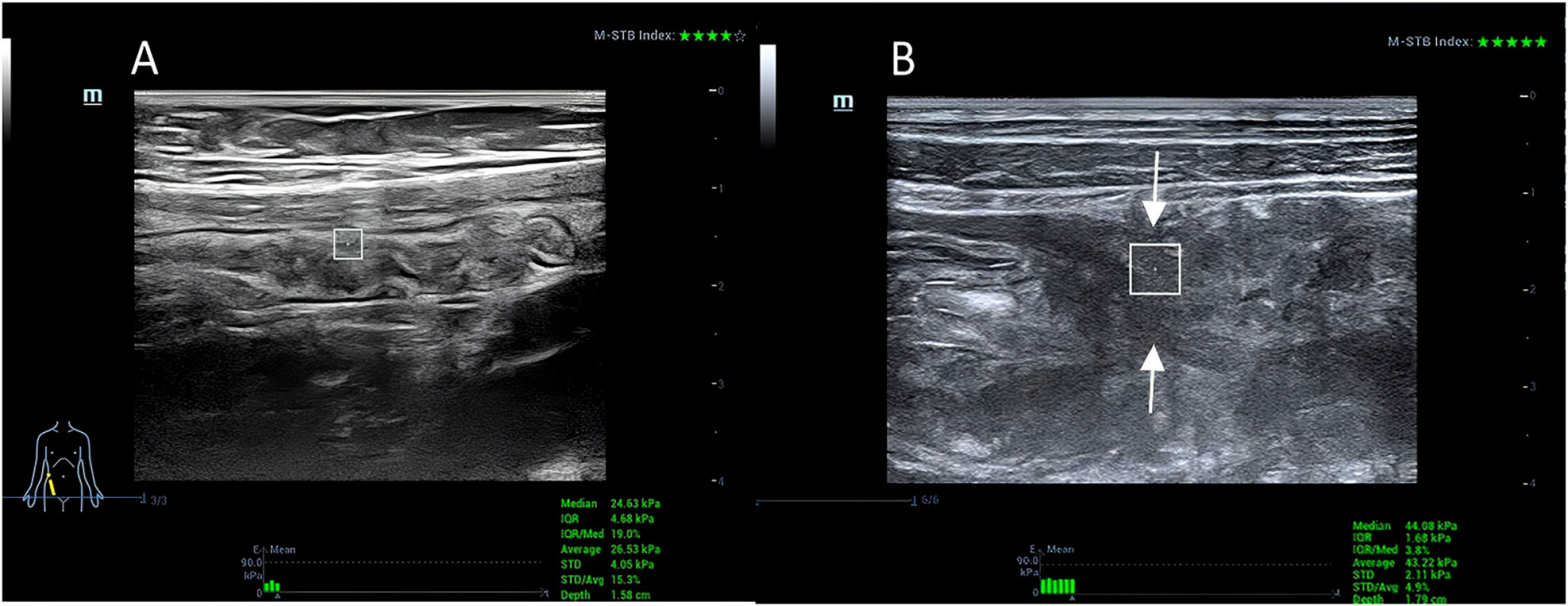
**A** Shows a representative elastography image of a non-stenotic intestinal wall with a shear wave velocity of 2.87 m/s. **B** Illustrates a stenotic site with a significantly higher shear wave velocity of 3.83 m/s, marked by an arrow indicating the stenotic zone

VTIQ measurements were acquired at the thickest segment of the intestinal wall (≥ 3 mm), with peristalsis avoided via real-time monitoring. Five consecutive readings were averaged. Image quality was confirmed via built-in QC. Representative elastography images are shown in Fig. 1 (panel A: non-stenotic wall; panel B: stenotic zone).

### Ultrasound image acquisition and analysis

After initial B-mode ultrasound screening to identify target bowel segments, VTIQ elastography was performed using a Mindray Resona R9T system with a 9L-4 linear transducer (4–9 MHz). All acquisitions followed standardized protocols, with key operational details as follows:

ROI selection: the thickest segment of the bowel wall was localized via multiplanar scanning (longitudinal and transverse views) to confirm the region of maximum thickness. Segments with intraluminal gas or active peristalsis (assessed via real-time dynamic observation) were excluded to ensure tissue homogeneity. For VTIQ measurements, a 3 × 3 mm ROI was placed 1 mm below the mucosal layer (identified via B-mode layer differentiation). This placement focused on the muscularis propria while avoiding serosal fat and adjacent extraintestinal tissues.

Peristalsis avoidance and measurement stability: all measurements were acquired during the quiescent phase of intestinal peristalsis. Each patient was monitored for at least 2 min to allow for peristalsis cessation, with real-time B-mode guidance to confirm static tissue status. Five consecutive measurements were recorded for each ROI; values deviating > 10% from the mean of the five measurements were excluded. The final result for each ROI was calculated as the mean of the remaining three stable measurements (with ≥ 3 valid values retained) to minimize variability from transient peristalsis.

### Gold standard

Intestinal stenosis was assessed using the Simplified Endoscopy for Crohn’s Disease (SES-CD) scoring system. SES-CD scores from enteroscopy served as the gold standard for stenosis severity. The SES-CD scoring system, derived from enteroscopy, served as the gold standard for assessing intestinal stenosis severity in our study. This widely accepted scoring system provides a comprehensive evaluation of mucosal changes and is considered a reliable reference for comparing the diagnostic accuracy of VTIQ.

### Validation by radiology consultants

To ensure the reliability and validity of our results, all VTIQ examinations were reviewed and validated by two additional radiology consultants from our department. The first consultant is a senior physician with 30 years of experience in radiology, and the second consultant is a physician with 14 years of experience in the field. Both consultants independently reviewed the ultrasound images and data, confirming the findings and ensuring consistency in the interpretation of the results.

### Statistical analysis

Data were analyzed using SPSS version 26.0 and R 4.4.1. Quantitative data were expressed as mean ± standard deviation or median and interquartile range (IQR). Categorical variables were described as frequencies or percentages. Comparisons between groups were made using an independent samples *t*-test or a Mann–Whitney *U*-test. Risk factors were identified using logistic regression analysis. For multivariate logistic regression analysis, potential predictors were initially screened via univariate analysis. Variables with a *p*-value < 0.1 in univariate analysis were included in the multivariate model. This threshold was chosen to balance statistical rigor and clinical relevance: Given the moderate sample size (*n* = 63) in our study, a relatively lenient cutoff (*p* < 0.1 rather than the stricter *p* < 0.05) was adopted to minimize the risk of excluding potentially important confounding factors that might have clinical significance but failed to reach the stricter statistical significance due to limited statistical power. This approach is consistent with common practices in observational studies with small-to-moderate sample sizes, aiming to retain variables that could exert meaningful effects on the outcome (stenosis detection) while avoiding overfitting the model by including an excessive number of predictors. Diagnostic accuracy metrics (sensitivity, specificity, PPV, NPV) were calculated against SES-CD as the reference standard. Confidence intervals (CIs) were computed using exact binomial methods.

## Results

To clarify the analytical logic of this study, the results are presented in three progressive steps: first, comparing baseline clinical and ultrasound characteristics between patients with and without intestinal stenosis to identify potential differences; second, using univariate and multivariate logistic regression analyses to screen for independent risk factors associated with stenosis; and finally, evaluating the diagnostic performance of individual and combined ultrasound indices through receiver operating characteristic (ROC) curve analysis and comparison with the gold standard (SES-CD).

### Baseline data comparison

No significant differences were found in gender, age, C-reactive protein (CRP), or erythrocyte sedimentation rate (ESR) (Table 1). Intestinal bowel ultrasound stenosis assessment score (IBUS-SAS) (*p* = 0.036), shear wave velocity (*p* < 0.001), and Young’s modulus values (*p* < 0.001) exhibited significant differences (Table 2).

**Table 1:** Clinical data comparisons between patients with and without intestinal stenosis

Group	Non-stenosis	Stenosis	Total	*p*
	(*N* = 32)	(*N *= 31)	(*N* = 63)	
Gender				0.888
Female	15 (46.88%)	13 (41.94%)	28 (44.44%)	
Male	17 (53.12%)	18 (58.06%)	35 (55.56%)	
Age	26.50 [18.50; 35.00]	31.00 [26.00; 37.00]	30.00 [22.00; 35.50]	0.173
Colonoscopy(SES-CD)				0.302
Active	25 (78.12%)	28 (90.32%)	53 (84.13%)	
Remission	7 (21.88%)	3 (9.68%)	10 (15.87%)	
CRP	2.73 [0.68; 11.46]	4.72 [1.38; 13.79]	3.19 [0.90; 11.46]	0.145
ESR	9.00 [4.50; 22.00]	9.00 [4.00; 26.00]	9.00 [4.00; 22.50]	0.777

**Table 2:** Ultrasound imaging data comparisons between patients with and without intestinal stenosis

Group	Non-stenosis	Stenosis	Total	*p*
	(*N* = 32)	(*N* = 31)	(*N* = 63)	
Inflammatory mesenteric fat				0.412
Absent	13 (40.62%)	9 (29.03%)	22 (34.92%)	
Uncertain	6 (18.75%)	4 (12.90%)	10 (15.87%)	
Present	13 (40.62%)	18 (58.06%)	31 (49.21%)	
Color Doppler flow grade				0.820
Absent	3 (9.38%)	1 (3.23%)	4 (6.35%)	
Short signals	12 (37.50%)	11 (35.48%)	23 (36.51%)	
Long signals inside bowel	12 (37.50%)	14 (45.16%)	26 (41.27%)	
Long signals inside and outside bowel	5 (15.62%)	5 (16.13%)	10 (15.87%)	
Limberg classification				0.845
I	3 (9.38%)	2 (6.45%)	5 (7.94%)	
II	13 (40.62%)	10 (32.26%)	23 (36.51%)	
III	11 (34.38%)	14 (45.16%)	25 (39.68%)	
IV	5 (15.62%)	5 (16.13%)	10 (15.87%)	
Bowel wall stratification				0.662
Normal	15 (46.88%)	10 (32.26%)	25 (39.68%)	
Uncertain	3 (9.38%)	5 (16.13%)	8 (12.70%)	
Focal < = 3 cm	8 (25.00%)	10 (32.26%)	18 (28.57%)	
Extensive > 3 cm	6 (18.75%)	6 (19.35%)	12 (19.05%)	
Bowel wall thickness (mm)	6.50 [5.35;8.00]	7.60 [5.30;9.70]	6.90 [5.35;8.70]	0.201
IBUS-SAS	54.62 ± 30.38	70.20 ± 26.99	62.29 ± 29.59	0.036
Shear wave velocity (m/s)	2.31 ± 0.79	3.35 ± 0.72	2.82 ± 0.91	< 0.001
Young’s modulus value (kPa)	19.65 [15.13; 29.50]	35.00 [23.50; 44.71]	25.00 [17.85; 38.00]	< 0.001

### Logistic regression analysis

#### Univariate analysis of intestinal stenosis in CD patients

The univariate analysis results indicate that among patients with CD, shear wave velocity, Young’s modulus value, and IBUS-SAS are significantly associated with the occurrence of intestinal stenosis (*p* < 0.05). (Table 3).

**Table 3:** Univariate logistic regression analysis

Variable	B	Standard Error	Wald	Degrees of Freedom	Significance	OR	OR (95% CI)	
Gender	0.2	0.508	0.155	1	0.693	1.222	0.451	3.306
Age	0.012	0.019	0.381	1	0.537	1.012	0.975	1.051
Colonoscopy (SES-CD)	−0.961	0.743	1.672	1	0.196	0.383	0.089	1.641
Bowel wall thickness mm	0.098	0.09	1.19	1	0.275	1.103	0.925	1.314
Inflammatory mesenteric fat	0.358	0.283	1.599	1	0.206	1.43	0.821	2.491
Color Doppler flow grade	0.224	0.312	0.515	1	0.473	1.251	0.679	2.304
Limberg classification	0.209	0.302	0.479	1	0.489	1.233	0.682	2.229
Bowel wall stratification	0.169	0.217	0.609	1	0.435	1.184	0.774	1.811
IBUS-SAS	0.019	0.009	4.222	1	0.04	1.019	1.001	1.038
Shear wave velocity (m/s)	1.828	0.483	14.329	1	0	6.219	2.414	16.022
Young’s modulus value (kPa)	0.098	0.027	12.853	1	0	1.103	1.045	1.164
CRP	−0.004	0.011	0.12	1	0.729	0.996	0.976	1.017
ESR	0.001	0.013	0.007	1	0.932	1.001	0.975	1.028

#### Multivariate analysis of intestinal stenosis in CD patients

In patients with CD, multivariate logistic regression analysis was conducted by including factors with a *p*-value less than 0.1 from the univariate analysis. The analysis revealed Shear wave velocity (odds ratio (OR) = 3.943, 95% CI: 1.426–10.905, *p* = 0.008), Young’s modulus value (OR = 1.079, 95% CI: 1.001–1.163, *p* = 0.046), and IBUS-SAS (OR = 1.033, 95% CI: 1.006–1.060, *p* = 0.015) were significant risk factors (Table 4).

**Table 4:** Multivariate logistic regression analysis

Variable	B	Standard error	Wald	Degrees of freedom	Significance	OR	OR (95% CI)	
IBUS-SAS	0.033	0.013	5.941	1	0.015	1.033	1.006	1.06
Shear wave velocity (m/s)	1.372	0.519	6.989	1	0.008	3.943	1.426	10.905
Young’s modulus value (kPa)	0.076	0.038	3.991	1	0.046	1.079	1.001	1.163

### ROC analysis

**Fig. 2:**
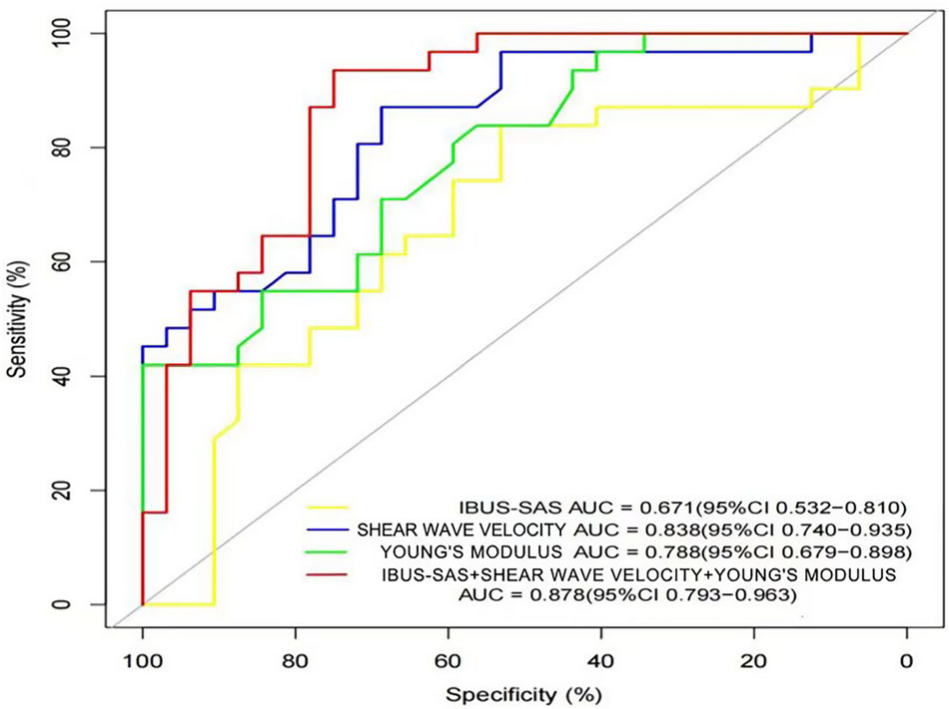
ROC curves for evaluating the diagnostic accuracy of individual ultrasound parameters (IBUS-SAS, shear wave velocity, Young’s modulus) and their combined model in detecting intestinal stenosis in CD, with SES-CD as the gold standard. Yellow line: IBUS-SAS (AUC = 0.671, 95% CI: 0.532–0.810). Green line: Young’s modulus (AUC = 0.788, 95% CI: 0.679–0.898). Blue line: Shear wave velocity (AUC = 0.838, 95% CI: 0.740–0.935). Red line: Combined model (IBUS-SAS + shear wave velocity + Young’s modulus, AUC = 0.878, 95% CI: 0.793–0.963). Higher AUC values indicate superior diagnostic performance

The diagnostic performance varied among modalities: IBUS-SAS showed moderate accuracy (AUC = 0.671, 95% CI: 0.55–0.79), while shear wave velocity achieved the highest AUC (0.838, 95% CI: 0.76–0.92), outperforming Young’s modulus (AUC = 0.788, 95% CI: 0.68–0.89). The combined model yielded an AUC of 0.878, indicating superior predictive performance. The comparative diagnostic performance of individual and combined parameters is illustrated in Fig. 2.

### Diagnostic accuracy against endoscopy

When using endoscopy (SES-CD) as the gold standard, ultrasound demonstrated: True positives: 22/31 stenosis cases correctly identified. False negatives: 9/31 stenosis cases missed. False positives: 0/32 non-stenosis cases misclassified. True negatives: 32/32 non-stenosis cases correctly ruled out (Table 5).

**Table 5:** Cross-tabulation of ultrasound vs endoscopy findings and diagnostic performance metrics

	Endoscopy:Stenosis (+)	Endoscopy:NonStenosis (−)	Row total	Metric	Formula	Value (95% CI)
US:Stenosis (+)	22 (TP)	0 (FP)	22	Sensitivity	TP/(TP + FN)	71.0% (52.0–85.8%)
US:Stenosis (−)	9 (FN)	32 (TN)	41	Specificity	TN/(TN + FP)	100% (89.1–100%)
Column total	31	32	63	PPV	TP/(TP + FP)	100% (84.6–100%)
				NPV	TN/(TN + FN)	78.0% (62.4–89.4%)
				Accuracy	(TP + TN)/(TP + TN + FP + FN)	85.7% (74.6–93.3%)

*TP* true positive, *FP* false positive, *FN* false negative, *TN* true negative

### Imaging characteristics of CD

**Fig. 3:**
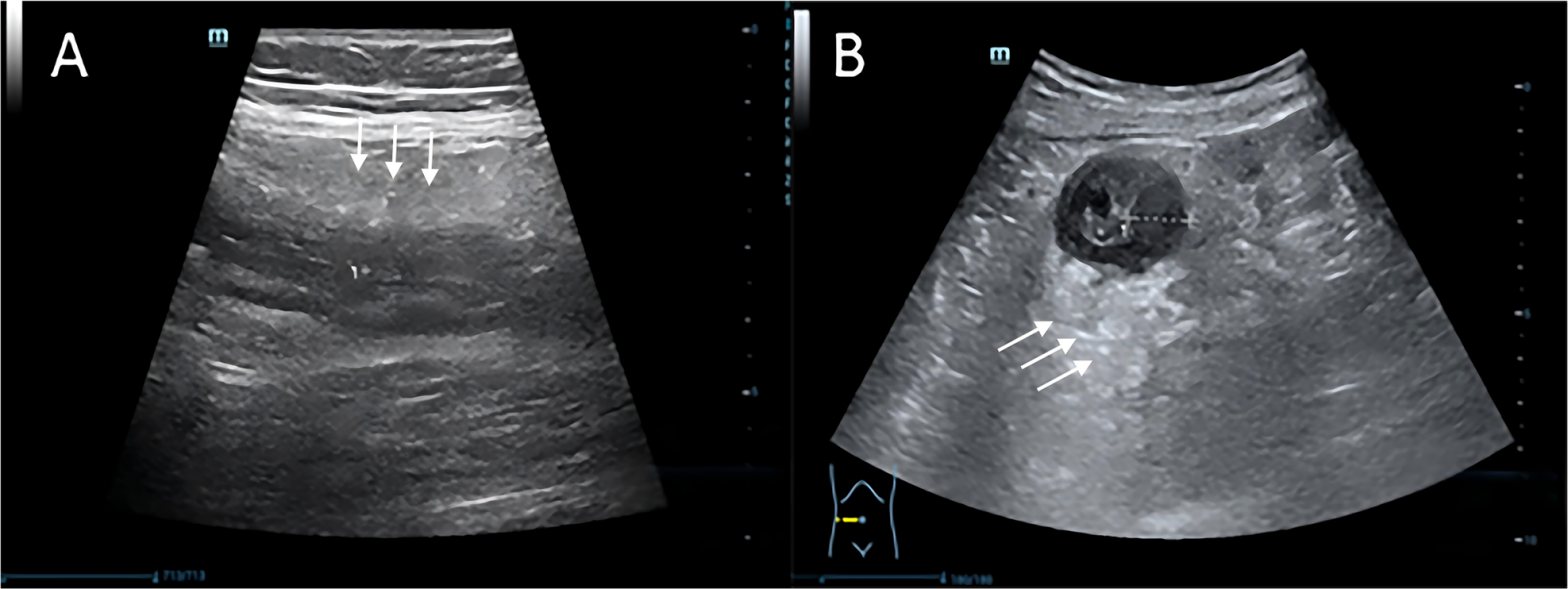
Increased echogenicity of the perienteric fat tissue (fat creeping sign) in CD patients. **A** Longitudinal section. **B** Transverse section (white arrows indicate fat creeping)

**Fig. 4:**
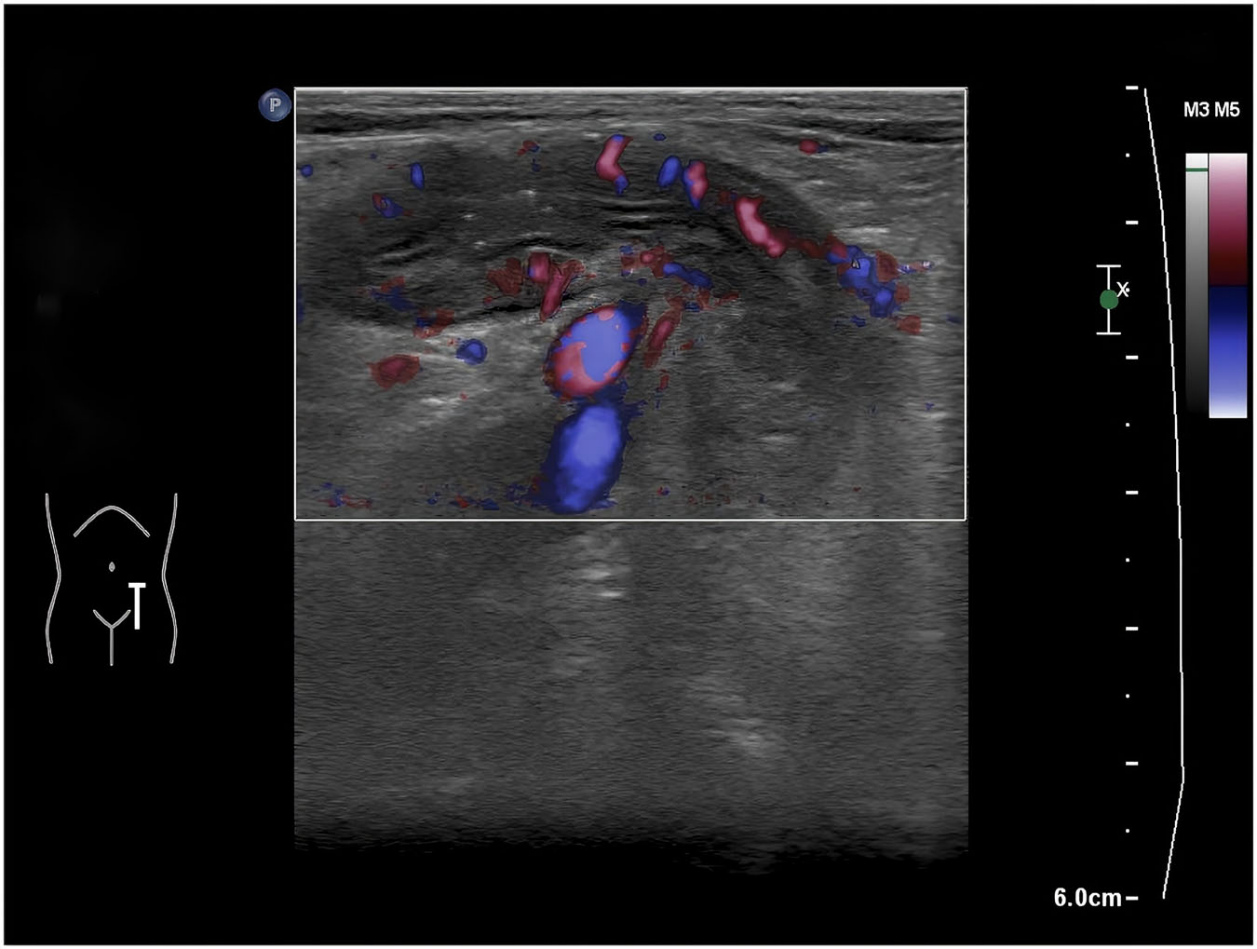
Blood flow grading by Limberg classification. This figure showed Grade IV: mesentery-extending flows

Typical ultrasound features of CD were observed in all patients. The fat creeping sign, characterized by hyperechoic perienteric fat tissue surrounding the affected intestinal wall, was present in 31 cases (49.2%) (Fig. 3). Additionally, blood flow signals within the intestinal wall were graded according to the Limberg classification (Fig. 4).

## Discussion

CD is a chronic inflammatory condition characterized by diverse manifestations and a high risk of complications such as fibrosis, strictures, and fistulas. Effective assessment of disease activity and control of inflammation are crucial for improving patient outcomes [9]. Current imaging techniques, including magnetic resonance imaging (MRI) and computed tomography (CT), have limitations, particularly in patients with contraindications such as renal failure, pregnancy, or contrast agent allergies [10]. Transabdominal ultrasound, being radiation-free and non-invasive, offers a promising alternative for evaluating intestinal wall thickness, structure, and blood flow changes [11, 12].

### Clinical significance of VTIQ in CD diagnosis

The introduction of ultrasound elastography, particularly VTIQ, has revolutionized the assessment of tissue stiffness in CD. Our study demonstrated that shear wave velocity and Young’s modulus values were significant predictors of intestinal stenosis, highlighting the potential of VTIQ in CD diagnosis. This is consistent with the application value of shear-wave elastography reported in related studies [13]. Specifically, the AUC of shear wave velocity for diagnosing intestinal stenosis in our study was 0.838, which is comparable to the findings of Chen et al [14] who reported an AUC of 0.81 for predicting CD disease progression using shear wave velocity. Compared to traditional methods, VTIQ provides quantitative data on tissue hardness, offering higher sensitivity and specificity, as supported by existing evidence [14, 15].

While ultrasound exhibited perfect specificity (100%) for ruling in stenosis, its 29% false-negative rate (9/31) warrants analysis of technical factors: first, 3 cases had deep pelvic lesions (upper rectum) where intraluminal gas could not be avoided, causing ROI measurement bias; second, 2 cases had incomplete cessation of intestinal peristalsis (despite 2-min monitoring), leading to unstable shear waves; third, 2 cases had stenosis mainly involving the mucosal layer, while ROI was placed in the muscularis propria (1 mm below the mucosa). Thus, negative results should be supplemented with an MRI if clinical symptoms (e.g., intestinal obstruction) persist.

IBUS-SAS, though less accurate than shear wave velocity (AUC 0.671 vs 0.838), remains an independent predictor of stenosis (OR = 1.033, *p* = 0.015), likely reflecting its composite assessment of bowel wall morphology and vascularity.

### Comparison with prior research and elucidation of incremental value

Our work on VTIQ-based assessment in CD builds closely on the foundational contributions of earlier studies, aiming to modestly address their limitations while extending their insights.

Dillman et al pioneered contrast-enhanced sonography (CEUS) for distinguishing CD-related fibrosis from inflammation in animal models, laying critical groundwork for ultrasound-based fibrosis evaluation [16]. However, their reliance on contrast agents (posing risks for renal-impaired CD patients), and preclinical design limited clinical translation. Our VTIQ approach seeks to complement this work: as a contrast-free, radiation-free technique, it is more broadly applicable to vulnerable subgroups, and our validation in 63 human patients showed shear wave velocity (SWV, AUC = 0.838) provides objective, clinically relevant stiffness data.

Calabrese et al advanced CD ultrasound by confirming interobserver agreement for conventional B-mode, yet highlighted variability in qualitative indices (e.g., bowel wall thickness) [17]. This challenges reliable stenosis diagnosis—especially distinguishing reversible inflammation from irreversible fibrosis. We attempted to mitigate this by integrating VTIQ’s quantitative parameters (SWV AUC = 0.838, Young’s modulus AUC = 0.788) with standardized protocols (e.g., daily phantom calibration). These metrics outperformed the qualitative IBUS-SAS (AUC = 0.671) in our cohort, offering a potential path to reduce operator bias.

Giannetti et al validated real-time elastography’s feasibility in CD, a key step toward non-invasive stiffness assessment [18]. Their semi-quantitative color grading, however, introduced subjectivity. Our VTIQ work supplements this by providing numerical stiffness values (e.g., SWV in m/s) and built-in QC. Importantly, we linked these metrics to stenosis—an unmet clinical need—via multivariate analysis, which identified SWV (OR = 3.943, *p* = 0.008) as an independent risk factor, moving beyond feasibility to actionable endpoints.

We emphasize these are incremental advances, not revolutionary changes. Our study owes much to these preceding works, and we view it as a small step toward refining ultrasound’s role in CD management.

### Comparison with other imaging techniques

Traditional imaging techniques such as CT and MRI have been widely used for CD diagnosis. However, CT involves radiation exposure, which poses risks for vulnerable populations such as pregnant women or patients requiring repeated imaging [4]; MRI, while radiation-free, is costly, time-consuming, and may be contraindicated in patients with renal insufficiency (due to gadolinium contrast risks) or metal implants [5]. In contrast, VTIQ addresses these limitations: its radiation-free nature eliminates fetal exposure risks in pregnant patients, and it requires no contrast agents, making it safe for those with renal impairment. Moreover, its real-time accessibility allows for rapid assessment in urgent clinical scenarios (e.g., acute CD flares). Additionally, VTIQ provides quantitative data on tissue stiffness, which is not available with conventional ultrasound or endoscopy [19]. This capability allows for a more comprehensive evaluation of disease activity and fibrosis, which is essential for guiding treatment decisions.

### Potential impact on patient management

The ability of VTIQ to quantitatively assess tissue stiffness has significant implications for patient management. For example, patients with high shear wave velocity values may benefit from early intervention to prevent the progression of fibrosis and strictures. Conversely, patients with low stiffness values may be candidates for less aggressive treatment regimens, reducing the risk of unnecessary side effects [20]. Furthermore, VTIQ can be used to monitor disease progression and treatment response over time, providing valuable information for adjusting therapeutic strategies. In terms of clinical practice, the combined model (AUC = 0.878) offers a practical workflow for stenosis assessment: First, initial screening: when shear wave velocity > 3.35 m/s (mean of stenosis group) and IBUS-SAS > 70 points (mean of stenosis group), specificity reaches 100% (Table 5), which can reduce unnecessary endoscopic procedures—particularly valuable for patients with contraindications to endoscopy (e.g., contrast allergy, pregnancy). Second, recheck: for cases with negative single-index results but high clinical suspicion of stenosis, the combined model’s NPV (78%) outperforms individual indices, minimizing the risk of missed diagnosis. Third, for obese patients (BMI > 28 kg/m^2^), moderate abdominal compression before scanning is recommended to reduce fat layer interference, and the probe frequency should be adjusted to 4–6 MHz to enhance penetration. For patients with deep pelvic or rectal lesions, the lateral or lithotomy position can be adopted, and endorectal ultrasound can be used for auxiliary positioning if necessary to ensure the ROI (3 × 3 mm) is accurately placed at the thickest part of the lesion, reducing measurement bias caused by bone and gas blocks. This stepwise approach enhances the utility of VTIQ in personalized CD management.

### Limitations and future directions

Despite its advantages, the accuracy of ultrasound imaging can be influenced by factors such as lesion location, tissue thickness, and operator skill. For instance, in obese patients or those with lesions in the rectum or pelvic segments, the diagnostic accuracy may be reduced, leading to potential false-positive or false-negative results [17]. Future studies should focus on optimizing imaging protocols and improving operator training to enhance diagnostic reliability. Future studies can be optimized as follows:

1. Adopt a multicenter collaborative design with strictly unified inclusion criteria and operational protocols, ensuring samples cover patients with diverse BMI ranges (especially those with BMI > 28), lesion locations (e.g., pelvis and rectum), and disease stages. The sample size should be expanded to at least 200 cases to enhance statistical power and generalizability.
2. Develop ultrasound technologies adapted to complex anatomical conditions, such as low-frequency probes with high penetration for obese patients and ultra-high-frequency focused probes for deep lesions. Combine artificial intelligence algorithms to real-time correct fat or gas interference and automatically optimize image quality.
3. Establish a standardized training and QC system, including simulated operation assessments, regular phantom calibration (error controlled within 0.2 m/s), and cross-center image review to reduce inter-operator technical variability.
4. Integrate VTIQ with other modalities (e.g., MRI enterography, serum fibrosis markers) to construct a multi-dimensional diagnostic model, improving detection accuracy for complex cases (e.g., those with fistulas or severe fibrosis).

## Conclusion

This study identified IBUS-SAS, shear wave velocity, and Young’s modulus as independent risk factors for intestinal stenosis in CD, with shear wave velocity being the most accurate predictor. The combination of these parameters significantly improved diagnostic performance, suggesting that VTIQ technology has the potential to become a valuable tool in the management of CD. However, further high-quality, multicenter studies with larger sample sizes are needed to validate these findings and explore the broader clinical applications of VTIQ in inflammatory bowel disease.

In conclusion, VTIQ technology, particularly shear wave velocity, shows significant potential in the diagnosis and management of CD. Its ability to quantitatively assess intestinal stiffness offers a non-invasive alternative to traditional imaging methods, with implications for early intervention and personalized treatment strategies.

## Data Availability

The datasets generated and analyzed during the current study are available from the corresponding author on reasonable request. The raw data supporting the conclusions of this article will be made available by the authors, without undue reservation, to any qualified researcher. Additionally, any materials necessary for the replication of the study will be provided upon request.

## Abbreviations

AUC: Area under the curve
CD: Crohn’s disease
CI: Confidence interval
CRP: C-reactive protein
ESR: Erythrocyte sedimentation rate
IBUS-SAS: Intestinal bowel ultrasound stenosis assessment score
MI: Mechanical index
OR: Odds ratio
QC: Quality control
ROC: Receiver operating characteristic
ROI: Region of interest
SES-CD: Simplified endoscopy for Crohn’s disease
VTIQ: Virtual touch tissue imaging and quantification
